# Retrospective Observational Study of Patients With Subdural Hematoma Treated With Idarucizumab

**DOI:** 10.1089/neur.2023.0065

**Published:** 2023-11-09

**Authors:** Eiichi Suehiro, Hideyuki Ishihara, Yohei Kogeichi, Tsunenori Ozawa, Koichi Haraguchi, Masaru Honda, Yumie Honda, Makoto Inaba, Ryusuke Kabeya, Naoaki Kanda, Kenta Koketsu, Nobukuni Murakami, Hidetoshi Nakamoto, Kotaro Oshio, Kuniyasu Saigusa, Takashi Shuto, Shuichi Sugiyama, Kenji Suzuyama, Tsuguaki Terashima, Mitsuharu Tsuura, Mitsutoshi Nakada, Hitoshi Kobata, Toshio Higashi, Nobuyuki Sakai, Michiyasu Suzuki

**Affiliations:** ^1^ Department of Neurosurgery, International University of Health and Welfare, School of Medicine, Narita, Japan.; ^2^ Department of Neurosurgery, Yamaguchi University School of Medicine, Ube, Japan.; ^3^ Department of Neurosurgery, Nara Medical University, Kashihara, Japan.; ^4^ Department of Neurosurgery, Sannocho Hospital, Sanjo, Japan.; ^5^ Department of Neurosurgery, Hakodate Shintoshi Hospital, Hakodate, Japan.; ^6^ Department of Neurosurgery, Shunan Memorial Hospital, Kudamatsu, Japan.; ^7^ Department of Neurosurgery, Tokai University School of Medicine, Isehara, Japan.; ^8^ Department of Neurosurgery, Saiseikai Yokohamashi Tobu Hospital, Yokohama, Japan.; ^9^ Department of Neurosurgery, Ichinomiya Municipal Hospital, Ichinomiya, Japan.; ^10^ Department of Neurology, Imamura General Hospital, Kagoshima, Japan.; ^11^ Department of Neurological Surgery, Chiba Hokuso Hospital, Nippon Medical School, Inzai, Japan.; ^12^ Department of Neurosurgery, Japanese Red Cross Society, Kyoto Daini Hospital, Kyoto, Japan.; ^13^ Epilepsy Center, TMG Asaka Medical Center, Asaka, Japan.; ^14^ Department of Neurosurgery, Kawasaki Municipal Tama Hospital, Kawasaki, Japan.; ^15^ Department of Neurosurgery, Tokyo Bay Urayasu Ichikawa Medical Center, Urayasu, Japan.; ^16^ Department of Neurosurgery, Yokohama Rosai Hospital, Yokohama, Japan.; ^17^ Department of Neurosurgery, Yamaguchi Rosai Hospital, Sanyoonoda, Japan.; ^18^ Department of Neurosurgery, Karatsu Red Cross Hospital, Karatsu, Japan.; ^19^ Advanced Critical Care Center, Aichi Medical University Hospital, Nagakute, Japan.; ^20^ Department of Neurosurgery, Japanese Red Cross Society Wakayama Medical Center, Wakayama, Japan.; ^21^ Department of Neurosurgery, Kanazawa University Hospital, Kanazawa, Japan.; ^22^ Osaka Mishima Emergency Critical Care Center, Takatsuki, Japan.; ^23^ Department of Neurosurgery, Fukuoka University Chikushi Hospital, Chikushino, Japan.; ^24^ Department of Neurosurgery, Kobe City Medical Center General Hospital, Kobe, Japan.; ^25^ Department of Advanced ThermoNeuroBiology, Yamaguchi Graduate School of Medicine, Ube, Japan.

**Keywords:** dabigatran, exacerbation, idarucizumab, reversal therapy, traumatic brain injury

## Abstract

Use of anticoagulants is increasing with the aging of societies. The safe first-line drug is likely to be a direct oral anticoagulant (DOAC), but outcomes of treatment of traumatic brain injury (TBI) with anticoagulants are uncertain. Therefore, we examined the clinical effect of idarucizumab as reversal therapy in elderly patients with TBI who were treated with dabigatran. A retrospective multi-center observational study was performed in patients ≥65 years of age who developed acute traumatic subdural hematoma during treatment with dabigatran and underwent reversal therapy with idarucizumab. The items examined included patient background, neurological and imaging findings at arrival, course after admission, complications, and outcomes. A total of 23 patients were enrolled in the study. The patients had a mean age of 78.9 years. Cause of TBI was fall in 60.9% of the subjects. Mean Glasgow Coma Scale score at arrival was 8.7; anisocoria was present in 31.8% of cases. Exacerbation of consciousness was found in 30.4%, but only in 13.3% of subjects treated with idarucizumab before consciousness and imaging findings worsened. Dabigatran was discontinued in 81.8% of cases after hematoma development, with a mean withdrawal period of 12.1 days. The favorable outcome rate was 21.7%, and mortality was 39.1%. In multi-variate analysis, timing of idarucizumab administration was associated with a favorable outcome. There were ischemic complications in 3 cases (13.1%), and all three events occurred ≥7 days after administration of idarucizumab. These findings suggest that in cases that develop hematoma during treatment with dabigatran, it is important to administer idarucizumab early and restart dabigatran after conditions stabilize.

## Introduction

Japan is the first nation to become a hyperaging society and may serve as a role model for the design of therapeutic strategies for elderly patients. Our focus is on countermeasures for the prevention of traumatic brain injury (TBI) in elderly persons. The Japan Neurotrauma Data Bank Project 2015 (P2015) found that elderly patients with TBI attributable to a fall were likely to develop acute subdural hematoma and be asymptomatic in the early stage, and that treatment with anticoagulants frequently resulted in exacerbation.^1^ Similar cases have been reported in the United States.^2^ To avoid this pathology, guidelines recommend the discontinuation of anticoagulants and treatment with reversal drugs for patients with antithrombotic-related intracranial hemorrhage (ICH).^3^ However, the effects of reversal drugs in patients with ICH who are receiving anticoagulants are uncertain and definite evidence is lacking.^4–6^

Idarucizumab is a reversal drug specific to dabigatran and was approved in Japan in 2016. The rate of use of dabigatran among anticoagulants is relatively low in Japan, at ∼7–9%.^7,8^ However, dabigatran was the only anticoagulant with a specific reversal agent during the study period because Andexanet alfa was not yet available in Japan. Based on this background, idarucizumab was selected for evaluation of pharmacological reversal of anticoagulants by a reversal drug. In the current study, we examined the effect of idarucizumab in patients with TBI who were given dabigatran based on the current use of idarucizumab and past data from the Japan Neurotrauma Data Bank.

## Methods

A retrospective cohort study was performed in patients ≥65 years who had traumatic acute subdural hematoma while taking dabigatran (a direct thrombin inhibitor) and received idarucizumab as reversal therapy between September 2016 and December 2021. Criteria for patient enrollment were age 65 years or older with TBI, including findings of acute subdural hematoma on head computed tomography (CT) regardless of severity, treatment with daily dabigatran, and receiving idarucizumab 5 g intravenously during the acute phase of TBI. Physicians at each site made judgments on appropriate cases for the administration of idarucizumab for dabigatran reversal according to the Japanese guidelines for management of TBI and treatment.^9^ These guidelines state that anticoagulants and antiplatelet agents should be discontinued or reversed in patients with TBI who are taking antithrombotic drugs, but that careful consideration is required based on the underlying disease in each case.^9^

Patient data for TBI were provided by 23 collaborating clinical centers: Aichi Medical University Hospital, Dojinkai Shunan Memorial Hospital, Fukuoka University Chikushi Hospital, Hakodate Shintoshi Hospital, Ichinomiya Municipal Hospital, Japan Organization of Occupational Health and Safety Yokohama Rosai Hospital, Japanese Red Cross Kyoto Daini Hospital, Japanese Red Cross Society Karatsu Red Cross Hospital, Japanese Red Cross Wakayama Medical Center, Jiaikai Imamura General Hospital, Kanazawa University Hospital, Kawasaki Municipal Tama Hospital, Kobe City Medical Center General Hospital, Nara Medical University Hospital, Nippon Medical School Chiba Hokusoh Hospital, Osaka Mishima Emergency Medical Center, Saiseikai Yokohamashi Tobu Hospital, Sannocho Hospital, TMG Asaka Medical Center, Tokai University Hospital, Tokyo Bay Urayasu Ichikawa Medical Center, Yamaguchi Rosai Hospital, and Yamaguchi University Hospital. These participating institutions are actively involved in TBI care and follow the Japanese guidelines for management and treatment of TBI.^9^

There were 23 subjects in the study. Age, sex, mechanism of injury, Glasgow Coma Scale (GCS) on admission, pupillary abnormalities, head CT findings, acute neurological exacerbation (sudden drop of GCS by ≥2), acute exacerbation of imaging findings, details of anticoagulants, dabigatran discontinuation, timing of resumption of dabigatran, timing of idarucizumab administration, craniotomy, ischemic complications attributable to reversal therapy, bleeding complications attributable to resumption of dabigatran, and outcomes at discharge were examined in these subjects. The Glasgow Outcome Scale at discharge was used to assess outcome, with good recovery and moderate disability defined as a favorable outcome. The significance of categorical and nominal data was determined by chi-square test.

### Statistical analysis

Continuous data are presented as the mean ± standard deviation, and comparisons were performed with an unpaired *t*-test. Significance was assumed at *p* < 0.05. Clinical variables were used in multi-variate logistic regression analysis to identify independent predictors of a favorable outcome. A *p* value, 95% confidence interval (CI), and odds ratio (OR) are reported for significant variables in this analysis. All statistical analyses were conducted using SPSS software (version 22.0; IBM Corp., Armonk, NY). This project was approved by the institutional review board of the International University of Health and Welfare, School of Medicine (22-Nr-010). The requirement for individual patient consent was waived because of the observational nature of the study. This project has been registered with the University Hospital Medical Information Network (UMIN-CTR, No. 000047782) in Japan.

## Results

The background of the 23 subjects (73.9% males) is shown in Table 1. There was no patient with multiple trauma with an Abbreviated Injury Score (AIS) of ≥3, except for the head. The mean age was 78.9 ± 7.0 years, and the main cause of injury was fall (*n* = 14; 60.9%). The GCS on admission was 8.7 ± 3.5, and the prevalence of pupillary abnormality was 31.8%. In head CT, all cases had subdural hematoma. In brain herniation findings, disappearance or compression of the perimesencephalic cistern and midline shifts ≥5 mm were found in 14 (60.9%) and 18 (78.3%) cases, respectively. Deterioration of consciousness (GCS ≥2) in the acute phase occurred in 7 cases (30.4%), and deterioration of imaging findings occurred in 8 (34.8%).

**Table 1.** Characteristics of Patients

No. of patients	23
Age, years	78.9 ± 7.0
Sex (male)	73.9% (17/23)
Fall as cause of injury	60.9% (14/23)
Neurological findings on admission	
GCS	8.7 ± 3.5
Pupillary abnormalities	31.8% (7/22)
CT findings on admission	
Perimesencephalic cistern compression	60.9% (14/23)
Midline shift (≥5 mm)	78.3% (18/23)
Deterioration of consciousness in acute phase (GCS ≥2)	30.4% (7/23)
Deterioration of imaging findings in acute phase	34.8% (8/23)
Antithrombotic therapy	
Antiplatelet therapy	21.7% (5/23)
Anticoagulant therapy	100% (23/23)
Dabigatran withdrawal after injury	81.8% (18/22)
Resumption of dabigatran	50.0% (9/18)
No. of days post-injury before dabigatran resumption	12.1 ± 9.1
Administration of idarucizumab before deterioration of consciousness or imaging findings	65.2% (15/23)
Craniotomy	73.9% (17/23)
Ischemic complications after reversal therapy	13.0% (3/23)
Bleeding complications after dabigatran resumption	4.3% (1/23)
Outcome	
Favorable outcome	21.7% (5/23)
Mortality	39.1% (9/23)

Values are shown as mean ± standard deviation.

GCS, Glasgow Coma Scale; CT, computed tomography.

Before development of acute hematoma, all patients were taking dabigatran and 5 (21.7%) were taking concomitant antiplatelet agents (Table 1). Dabigatran was discontinued after hematoma development in 18 (81.8%) of 22 cases and restarted during hospitalization in 9 cases (50.0%). The anticoagulant withdrawal period post-injury was 12.0 ± 9.1 days. There were 15 subjects (65.2%) in which idarucizumab was administered before deterioration of consciousness or imaging findings (Table 1).

**FIG. 1.**
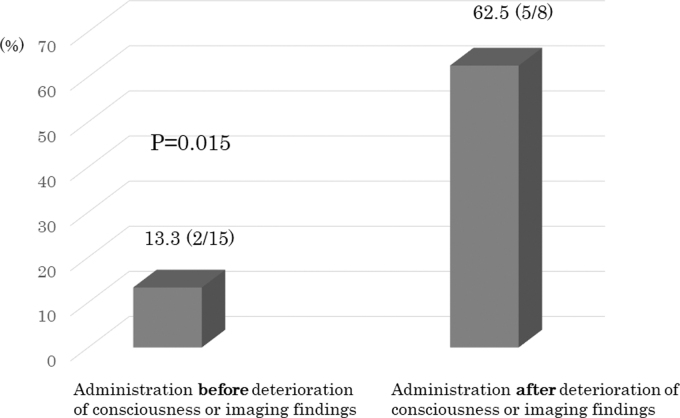
Rate of deterioration of consciousness in the acute phase depending on timing of idarucizumab administration as reversal therapy.

Comparisons were made between two groups of patients who received idarucizumab before or after worsening of their disease. Age (78.9 ± 7.0 vs. 78.8 ± 7.3 years), sex (80.0% vs. 62.5% male), rate of injury attributable to fall (53.3% vs. 75.0%), and GCS at hospital arrival (9.5 ± 3.7 vs. 7.3 ± 2.9) did not differ significantly between these groups (Table 2). However, the worst GCS in patients who received idarucizumab after disease progression was significantly lower than that in those who received reversal therapy before disease progression (4.8 ± 2.1 vs. 8.9 ± 3.7; Table 2). Rate of deterioration of consciousness in the acute phase was significantly lower (13.3%) when idarucizumab was administered before the start of deterioration of consciousness or imaging findings, compared to administration after this deterioration began (62.5%; Fig. 1).

**Table 2.** Patient Characteristics Divided into Groups Based on the Timing of Idarucizumab Administration

	Patients in which idarucizumab was administered before deterioration of consciousness or imaging findings	Patients in which idarucizumab was administered after deterioration of consciousness or imaging findings	*p* value
No. of patients	15	8	
Age, years	78.9 ± 7.0	78.8 ± 7.3	0.954
Sex (male)	80.0% (12/15)	62.5% (5/8)	0.363
Fall as cause of injury	53.3% (8/15)	75.0% (6/8)	0.311
Initial GCS	9.5 ± 3.7	7.3 ± 2.9	0.119
Worst GCS	8.9 ± 3.7	4.8 ± 2.1	0.008
Antithrombotic therapy			
Antiplatelet therapy	20.0% (3/15)	25.0% (2/8)	0.782
Anticoagulant therapy	100% (15/15)	100% (8/8)	—

Values are shown as mean ± standard deviation.

GCS, Glasgow Coma Scale.

Seventeen subjects (73.9%) underwent craniotomy (Table 1). Post-reversal ischemic and hemorrhagic complications after resumption of dabigatran occurred in 3 cases (13.0%) and 1 (4.3%) case, respectively (Table 1). Rate of a favorable outcome at discharge was 21.7%, and mortality was 39.1%. In a multi-variate model, only timing of idarucizumab administration was associated with a favorable outcome (OR = 0.008; 95% CI = 0.00–0.91; Table 3). The 3 patients with ischemic complications (Table 4) were between 79 and 90 years old, the disease was diffuse injury in all 3 cases, no cases had complications immediately after reversal therapy, all developed ischemic symptoms before resumption of dabigatran, and all had a poor outcome of severe disability.

**Table 3.** Multi-Variate Predictors of Favorable Outcome

Variable	OR (95% CI)	*p* value
Age	1.1 (0.89–1.34)	0.397
GCS	1.5 (0.82–2.63)	0.197
Concomitant antiplatelet agents	7.9 (0.10–665.08)	0.359
Timing of idarucizumab administration	0.0 (0.00–0.91)	0.045

GCS, Glasgow Coma Scale; OR, odds ratio; CI, confidence interval.

**Table 4.** Characteristics of Patients With Ischemic Complications

Age (years)	Sex	Mechanism of injury	GCS on admission	CT findings (TCDB classification)	Concomitant use of antiplatelet drugs	Onset of ischemic complications after reversal therapy (days)	Period after injury to resumption of dabigatran (days)	GOS at discharge
90	Male	Traffic accident	6	Diffuse injury IV	No	28	N/A	SD
80	Male	Fall	12	Diffuse injury II	No	7	18	SD
79	Female	Abuse	15	Diffuse injury II	Yes	7	8	SD

GCS, Glasgow Coma Scale; CT, computed tomography; TCDB, Traumatic Coma Data Bank; GOS, Glasgow Outcome Scale; SD, severe disability.

## Discussion

The aging society in many countries has led to an increased proportion of TBIs in elderly persons, and there is a need to recognize the characteristics of TBI in this patient population. Rate of TBI attributable to traffic accidents is relatively low and that attributable to fall is high.^10^ Because the subdural space is enlarged because of brain atrophy, the mobility of the cerebral hemispheres increases and the bridging veins are easily damaged.^10^ As a result, acute subdural hematoma is likely to occur, but symptoms rarely appear immediately after the injury and symptoms often worsen in a delayed manner.^10^ Although the frequency of extracranial injury and systemic complications is lower in older than in younger persons, extracranial injury and systemic complications are risk factors for poor outcomes.^11^ In addition, elderly persons have a high prevalence of arteriosclerosis and atrial fibrillation, and a high rate of oral administration of antiplatelet drugs and anticoagulants.^10^ In recent anticoagulant therapy for non-valvular atrial fibrillation, direct oral anticoagulants (DOACs) are used as the first-line drug, rather than warfarin. DOACs inhibit systemic and cerebral embolism more effectively or similarly to warfarin and significantly reduce hemorrhagic stroke; therefore, the safety of these drugs is apparent.^12–15^

Idarucizumab was approved in Japan in November 2016 and is used in reversal therapy as a specific neutralizer to eliminate the antithrombin activity of dabigatran.^16,17^ The reversal effect of idarucizumab on dabigatran is strong and rapid, which facilitates immediate coagulation, with a low risk for embolism.^18^ However, definite evidence of a clinical effect of idarucizumab in patients with ICH given dabigatran remains to be established. Therefore, we examined the use and effects of idarucizumab in Japan, including the timing of administration of reversal drugs, in TBI patients receiving idarucizumab who were treated with dabigatran.

Mean age of subjects in the current study was 78.9 years, which suggests that patients treated with idarucizumab in Japan tend to be elderly. Many were injured because of a fall, which was similar to the cases in the previous study.^1^ Findings at arrival in the current versus previous study (GCS score 8.7 vs. 10.5; pupillary defect ratio 31.8% vs. 20.8%) suggested more severe injury in the current study.^1^

The reversal drugs used in the previous study were mostly fresh frozen plasma and vitamin K,^1^ which are common in normal medical practice. Newer reversal drugs have recently been introduced, including prothrombin complex products for warfarin, idarucizumab for dabigatran, and Andexanet alfa for factor Xa inhibitors, but these drugs are expensive. Thus, we speculate that the reason for the more severe cases in the current study is that physicians are unlikely to use idarucizumab for mild cases because of the cost. In addition, 73.9% of patients treated with idarucizumab underwent a craniotomy, which was higher than the rate of 50.9% in the previous study.^1^ Thus, idarucizumab was used more frequently for patients who underwent a craniotomy because of evidence of the benefits of the use of reversal drugs for this surgery, which justified the cost of idarucizumab.

Improved outcomes of patients with TBI who are treated with anticoagulants requires the prevention of worsening of symptoms attributable to hematoma expansion. In this respect, the need for early administration of reversal drugs after injury is recommended. Decompressive craniectomy early post-injury is beneficial for patients with acute subdural hematoma,^19^ and early administration of hemostatic tranexamic acid is effective.^20^ Thus, active therapy is required in the early treatment of TBI. Therefore, we investigated the effect of the early administration of idarucizumab before worsening of consciousness and imaging findings. Such early use of idarucizumab reduced the rate of symptom aggravation to 13.3%, which is extremely low. A report of the Japan Neurotrauma Data Bank showed that the rate of worsening in elderly patients with TBI who were not treated with antithrombotic drugs was 17.6%, whereas that in patients treated with anticoagulants was 30.1%.^1^ Thus, the current study suggests that use of idarucizumab before pathogenic aggravation in elderly patients with TBI treated with dabigatran can reduce the rate of aggravation to the level of that in cases not given anticoagulants. The importance of early administration of reversal drugs for antithrombotic drugs was confirmed.

Three of 23 patients (13.0%) had ischemic complications after use of idarucizumab. In comparison with the rate of 6.3–7.4% in the RE-VERSE AD Clinical Trial,^17^ the rate in this study is higher; however, the safety of idarucizumab is still apparent. It is noteworthy that onset of ischemic complications occurred 7, 7, and 28 days after injury, respectively, and all before readministration of dabigatran in all 3 cases. Previous reports have recommended restarting anticoagulants in patients with TBI who were previously treated with these agents because ischemic complications were reduced by readministration of anticoagulants after the condition stabilized.^21,22^ Ischemic complications peak ∼3 days after discontinuation of anticoagulants,^23^ which suggests that these agents should be restarted within 24–72 h after injury.

## Conclusion

We examined the use of idarucizumab in patients with TBI who were treated with dabigatran. Idarucizumab was mainly used for severe cases requiring craniectomy. Idarucizumab was found to reduce pathogenic aggravation if the reversal therapy was started before worsening of the level of consciousness and imaging findings. We also recommend early restart of anticoagulants for prevention of ischemic complications.

## Abbreviations Used

CI: confidence interval
CT: computed tomography
GCS: Glasgow Coma Scale
ICH: intracranial hemorrhage
OR: odds ratio
TBI: traumatic brain injury
